# Reliability of pressure measurements via balloon catheters is high: an evaluation of six esophageal catheters

**DOI:** 10.1186/cc12049

**Published:** 2013-03-19

**Authors:** S Walterspacher, L Isaak, HJ Kabitz, J Guttmann, S Schumann

**Affiliations:** 1 University Medical Center Freiburg, Germany

## Introduction

Reliable measurement of esophageal pressure is a prerequisite for analysis of respiratory system mechanics in spontaneous breathing patients. For that purpose, various types of balloon catheters exist that differ in material, size and shape. In physical models we studied the quality of pressure measurement via six different balloon catheters, three of them containing a second balloon for measurement of gastric pressure.

## Methods

Nine balloons of six esophageal catheters were investigated in three conditions: measurement of balloon pressure during initial inflations immediately after unpacking; measurement of static pressures at different filling volumes; and compliance estimation in physical models (27, 54, 90 ml/cmH_2_O) at different levels of superimposed pressure.

## Results

During the initial inflation most catheters showed pressure artifacts resulting from material adhesion. Those artifacts disappeared during following inflations. Static pressure measurements could be performed with an error below 1 cmH_2_O if the balloon was filled appropriately. Overfilling of the balloon resulted in larger errors only in two catheters. Compliance estimations resulted in errors below 1 ml/ cmH_2_O. Superimposed pressure had no relevant effect on compliance estimation.

## Conclusion

The reliability of pressure measurements and also of compliance estimation via the tested catheters is high. Only in two catheters was the filling volume a critical point for a precise measurement of pressure or for estimation of compliance. Immediately after unpacking, adhesion of the balloon material might prevent reliable pressure measurement, therefore before the first measurement overfilling of the balloon and retention of the excess gas seems strongly recommended.

